# Publisher Correction: Integrated exome and RNA sequencing of dedifferentiated liposarcoma

**DOI:** 10.1038/s41467-020-14680-8

**Published:** 2020-02-19

**Authors:** Makoto Hirata, Naofumi Asano, Kotoe Katayama, Akihiko Yoshida, Yusuke Tsuda, Masaya Sekimizu, Sachiyo Mitani, Eisuke Kobayashi, Motokiyo Komiyama, Hiroyuki Fujimoto, Takahiro Goto, Yukihide Iwamoto, Norifumi Naka, Shintaro Iwata, Yoshihiro Nishida, Toru Hiruma, Hiroaki Hiraga, Hirotaka Kawano, Toru Motoi, Yoshinao Oda, Daisuke Matsubara, Masashi Fujita, Tatsuhiro Shibata, Hidewaki Nakagawa, Robert Nakayama, Tadashi Kondo, Seiya Imoto, Satoru Miyano, Akira Kawai, Rui Yamaguchi, Hitoshi Ichikawa, Koichi Matsuda

**Affiliations:** 1 Laboratory of Genome Technology, Institute of Medical Science, University of Tokyo, Tokyo, 108-8639 Japan; 2 Division of Rare Cancer Research, National Cancer Center Research Institute, Tokyo, 104-0045 Japan; 3 Department of Orthopaedic Surgery, Keio University School of Medicine, Tokyo, 160-8582 Japan; 4 Laboratory of Sequence Analysis, Institute of Medical Science, University of Tokyo, Tokyo, 108-8639 Japan; 5 Department of Pathology and Clinical Laboratory, National Cancer Center Hospital, Tokyo, 104-0045 Japan; 6 Department of Orthopaedic Surgery, Faculty of Medicine, University of Tokyo, Tokyo, 113-8654 Japan; 7 Department of Clinical Genomics, National Cancer Center Research Institute, Tokyo, 104-0045 Japan; 8 Department of Musculoskeletal Oncology, National Cancer Center Hospital, Tokyo, 104-0045 Japan; 9 Department of Urology, National Cancer Center Hospital, Tokyo, 104-0045 Japan; 10 Department of Orthopaedic Surgery and Musculoskeletal Oncology, Tokyo Metropolitan Cancer and Infectious Diseases Center Komagome Hospital, Tokyo, 113-8677 Japan; 11 Department of Orthopaedic Surgery, Graduate School of Medical Sciences, Kyushu University, Fukuoka, 812-8582 Japan; 12 Kyushu Rosai Hospital, Kitakyushu, 800-0296 Japan; 13 Musculoskeletal Oncology Service, Osaka International Cancer Institute, Osaka, 541-8567 Japan; 14 Division of Orthopaedic Surgery, Chiba Cancer Center, Chiba, 260-8717 Japan; 15 Department of Rehabilitation Medicine, Nagoya University Graduate School of Medicine, Nagoya, 464-8601 Japan; 16 Department of Orthopaedic Surgery, Kanagawa Cancer Center, Yokohama, 241-8515 Japan; 17 Department of Musculoskeletal Oncology, National Hospital Organization Hokkaido Cancer Center, Sapporo, 003-0804 Japan; 18 Department of Orthopaedic Surgery, Teikyo University School of Medicine, Tokyo, 173-8606 Japan; 19 Department of Pathology, Tokyo Metropolitan Cancer and Infectious Diseases Center Komagome Hospital, Tokyo, 113-8677 Japan; 20 Department of Anatomic Pathology, Graduate School of Medical Sciences, Kyushu University, Fukuoka, 812-8582 Japan; 21 Division of Integrative Pathology, Jichi Medical University, Shimotsuke, 329-0498 Japan; 22 Laboratory for Cancer Genomics, RIKEN Center for Integrative Medical Sciences, Yokohama, 230-0045 Japan; 23 Laboratory of Molecular Medicine, Institiute of Medical Science, University of Tokyo, Tokyo, 108-8639 Japan; 24 Division of Health Medical Data Science, Institute of Medical Science, University of Tokyo, Tokyo, 108-8639 Japan; 25 Laboratory of DNA Information Analysis, Institute of Medical Science, University of Tokyo, Tokyo, 108-8639 Japan; 26 Division of Translational Genomics, National Cancer Center-Exploratory Oncology Research & Clinical Trial Center, Tokyo, 104-0045 Japan; 27 Laboratory of Clinical Genome Sequencing, Graduate School of Frontier Sciences, University of Tokyo, Tokyo, 108-8639 Japan

**Keywords:** Sarcoma, Sarcoma, Sarcoma, Cancer genomics, Cancer genomics

Correction to: *Nature Communications* 10.1038/s41467-019-13286-z, published online 12 December 2019.

The original version of this Article contained an error in Fig. 4, in which samples marked with blue dots were labelled with the suffix ‘WD’ instead of ‘DD’. This has now been corrected in both the PDF and HTML versions of the Article.

