# Supplementary material for: Integrated exome and RNA sequencing of dedifferentiated liposarcoma
Source: Nat Commun. 2019 Dec 12;10:5683. doi: 10.1038/s41467-019-13286-z (PMC6908635; doi:10.1038/s41467-019-13286-z)
Supplement: Supplementary file 1 — Supplementary Information [file 41467_2019_13286_MOESM1_ESM.pdf]

Supplementary Information

Integrated Exome and RNA Sequencing of Dedifferentiated Liposarcoma

Hirata et al.

Supplementary Figure 1

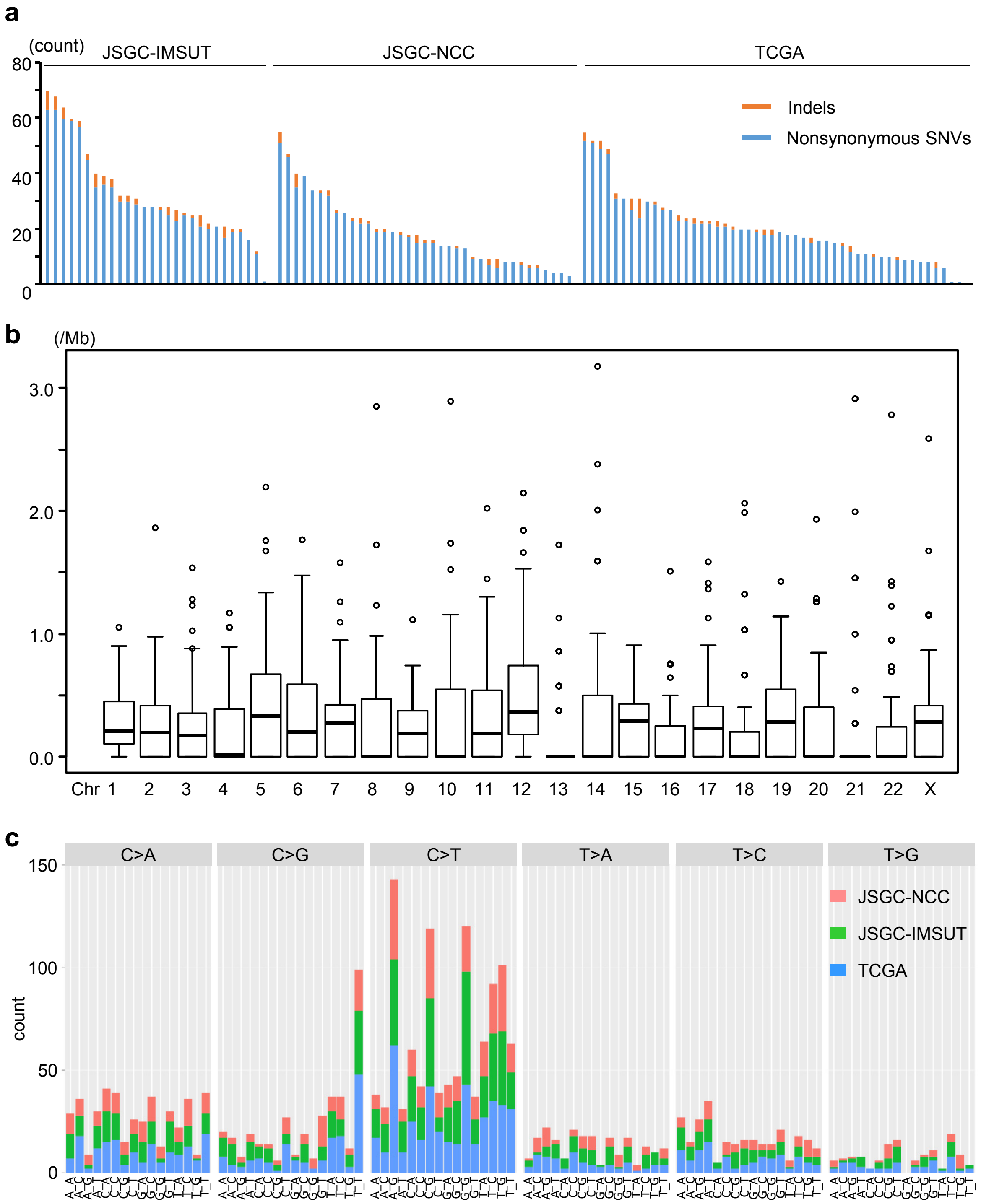

Supplementary Figure. 1 Somatic mutation profiles among three cohorts. **a** Frequencies of nonsynonymous SNVs and short Indels for each sample among three cohorts. Light blue and orange bars represent frequency of SNVs and Indels, respectively. **b** Nonsynonymous tumor mutation burden at each chromosome. The box signifies the upper and lower quartiles; the center bold line within the box, median; the upper and lower whiskers, upper and lower quartiles +/- interquartile ranges, respectively. **c** 96 substitution classification for DDLPS samples in the three cohorts. SNVs were classified by six base substitution patterns: C>A, C>G, C>T, T>A, T>C, T>G and by information on the bases immediately 5′ and 3′ to each mutated base.

Supplementary Figure 2

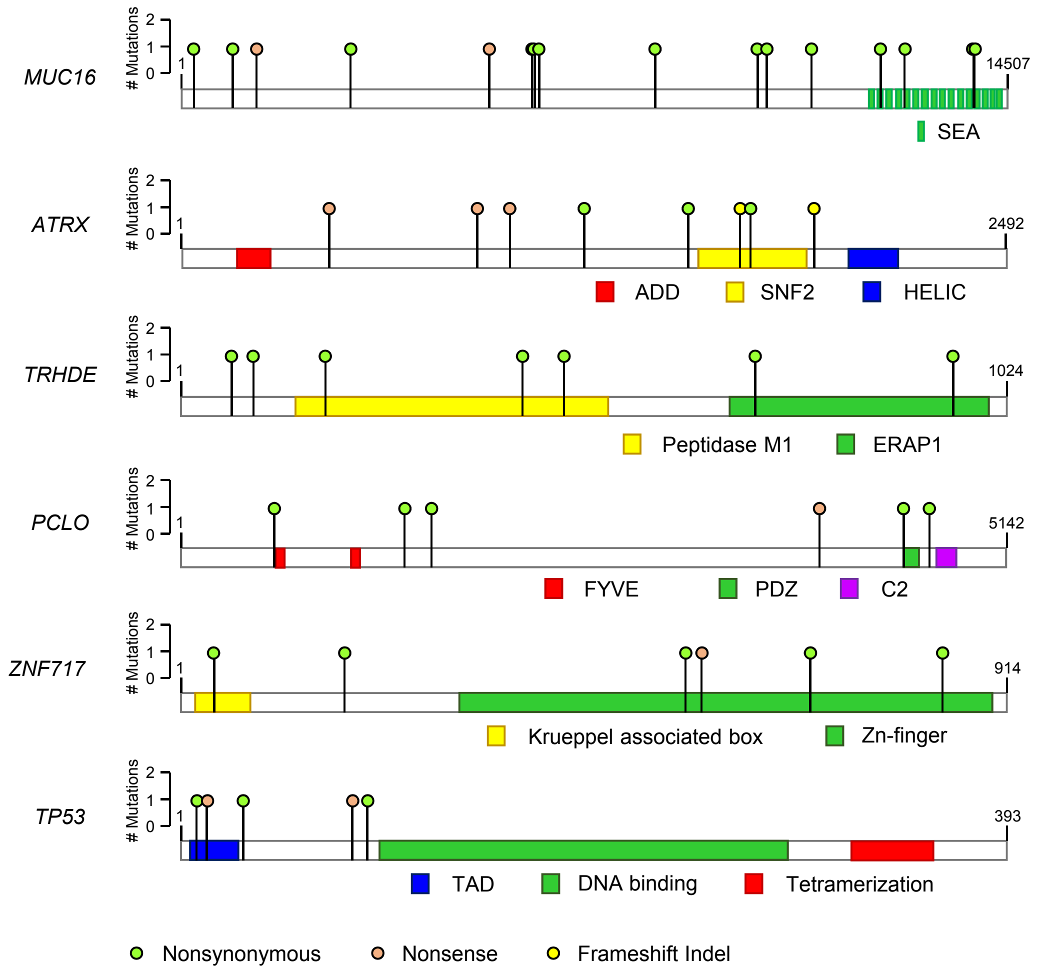

Supplementary Figure 2. Distribution of the somatic mutations identified in DDLPS. Distribution of the somatic mutations in *MUC16*, *ATRX, TRHDE, PCLO, TP53,* and *ZNF717*, which were identified in more than 5 samples of DDLPS. Lower box represents functional domains in each gene; vertical bars, mutation loci; circles, type of mutations.

Supplementary Figure 3

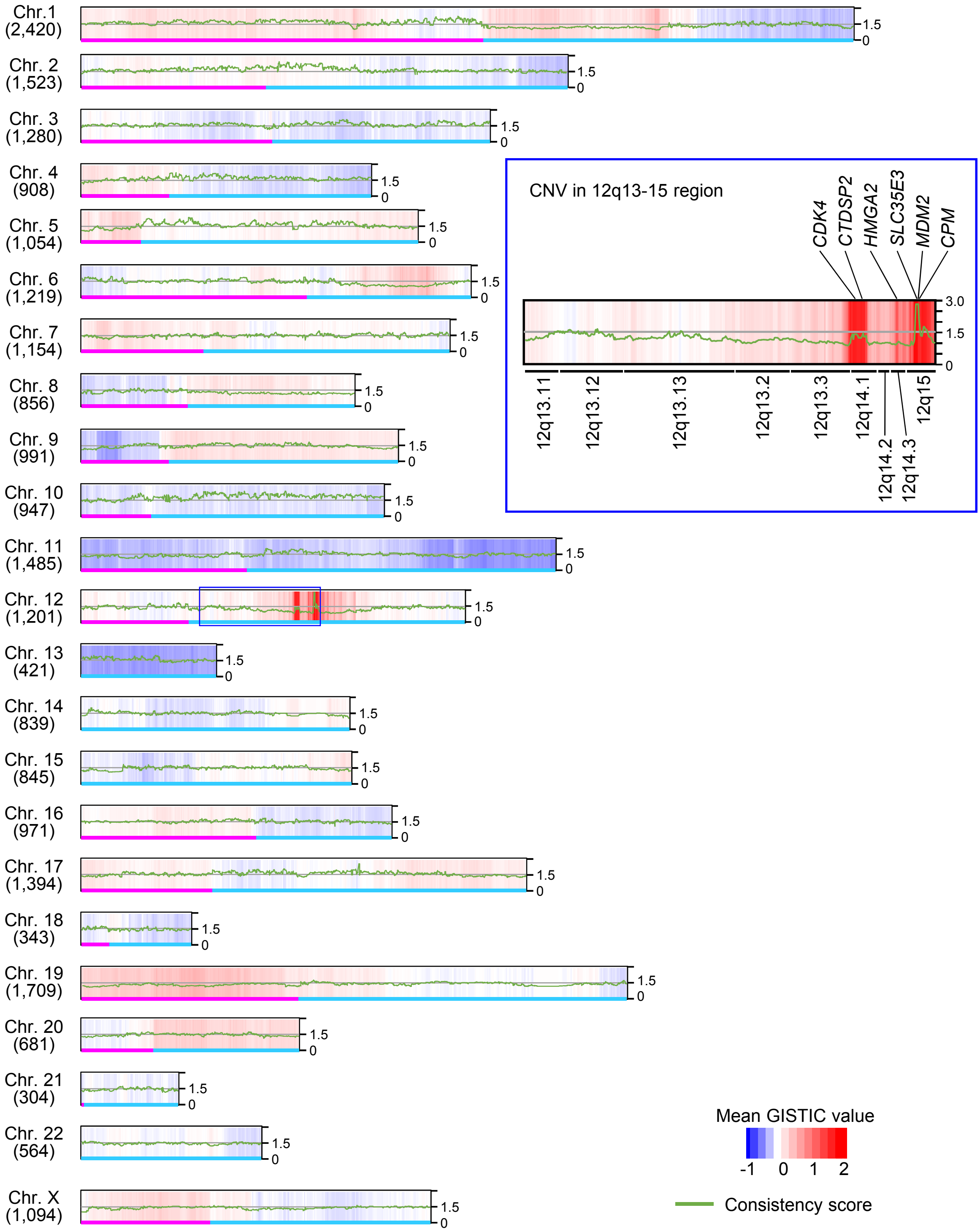

Supplementary Figure 3. Genome-wide SCNA pattern in DDLPS. Vertical heat-colored lines represent mean GISTIC value of each gene from 115 DDLPS samples. Numbers in the parentheses describes sum of the genes at each chromosome, subjected in this study (23,109 genes in total). Consistency score (green line) was produced by calculating inverse number of standard deviation of GISTIC value for each gene. Pink and blue bars indicate the short and long arms of each chromosome, respectively.

Supplementary Figure 4

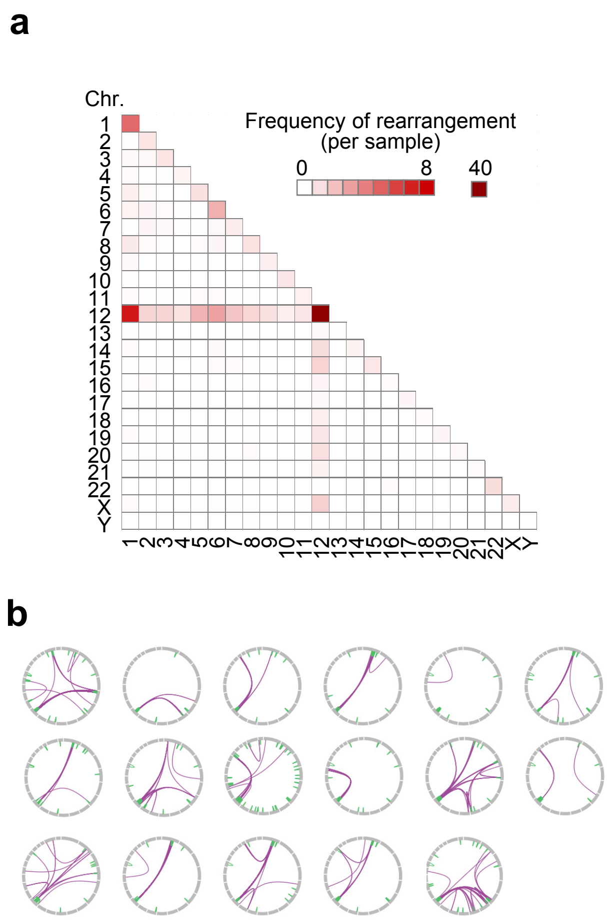

Supplementary Figure 4. Frequency of chromosomal rearrangements of DDLPS and WDLPS. **a** Frequency of inter- and intra-chromosomal rearrangements. Each box in the heatmap shows the mean frequency of inter- or intra- chromosomal rearrangement. The frequency of chromosomal rearrangements in each sample was determined using Genomon Fusion to analysis RNA sequencing results from 101 DDLPS tumors. **b,** Chromosomal rearrangements of 17 WDLPS samples. Circos plots represent the inter- (purple) and intra-(green) chromosomal rearrangements.

Supplementary Figure 5

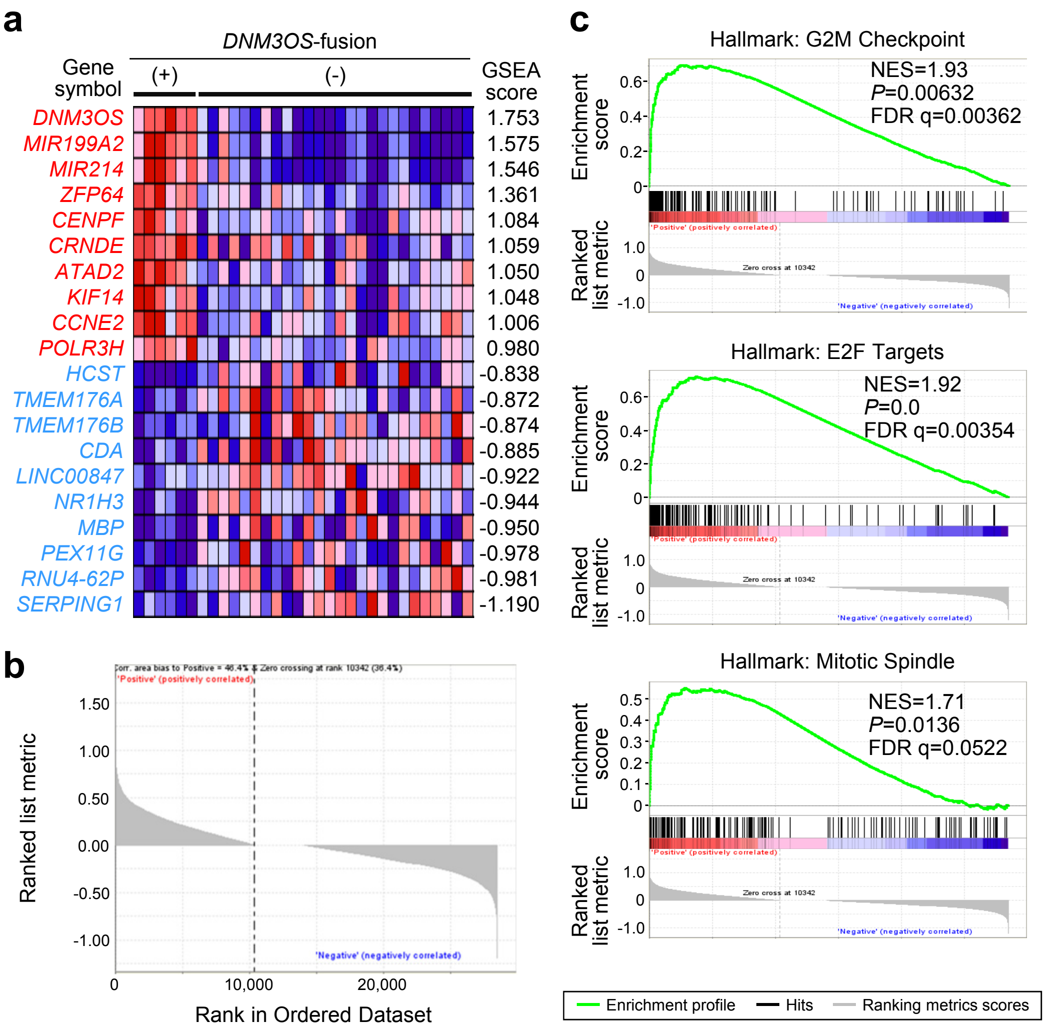

Supplementary Figure 5. Gene-set enrichment analysis (GSEA) between *DNM3OS*-fusion positive and negative DDLPS. **a** Heat map and gene list with the highest and lowest GSEA score in fusion positive DDLPS. Expression profiles were compared between *DNM3OS*-fusion positive (+) and negative (-) DDLPS from JSGC-NCC by GSEA. Genes with the 10 highest and lowest GSEA scores were listed. **b** Ranked list correlations between DDLPS with and without DNM3OS-fusion genes. GSEA ranking metrics scores of 28,449 genes were aligned in descending order. **c** Enrichment plots for gene sets enriched in DNM3OS-fusion positive DDLPS. Enrichment plots were depicted for gene sets with significant *P*-value (<0.05) and FDR q-value (<0.1) by GSEA analysis . GSEA analysis was conducted with Hallmark database by using RNA sequencing results from 32 (6 positive and 26 negative) JSGC-NCC samples.

Supplementary Figure 6

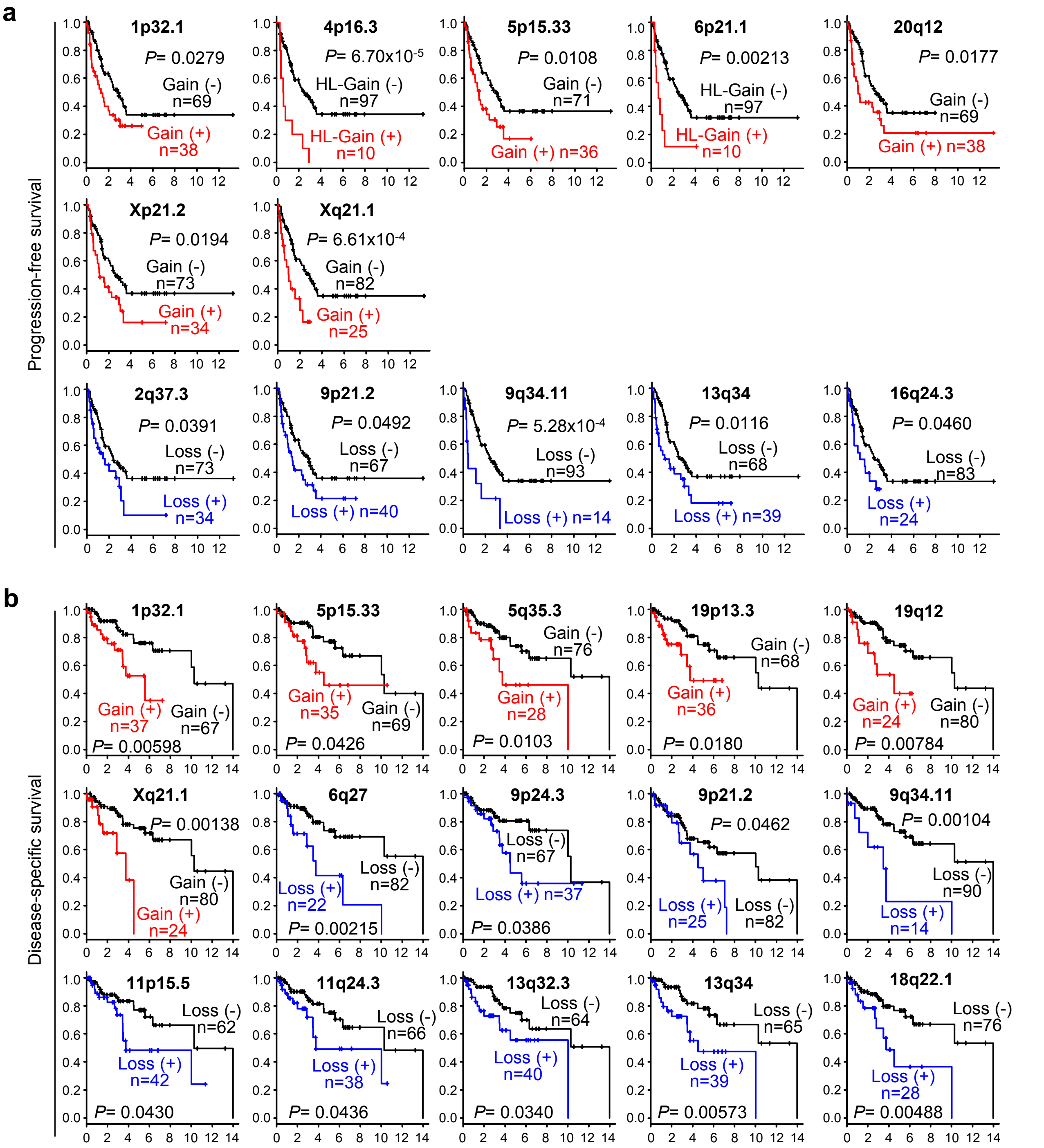

Supplementary Figure 6. Somatic copy number alterations (SCNAs) associated with progression-free and disease-specific survival. **a** Progression-free survivals by the status of 12 chromosome regions with gain and loss. **b** Disease-specific survivals by the status of 15 chromosome regions with gain and loss. Kaplan-Meier survival analysis was performed after stratifying patients with DDLPS by the status of recurrent SCNAs. SCNAs with gain were stratified by two criteria: high-level gain (HL-Gain) vs no HL-Gain (low-level or no gain), and Gain (high- or low-level gain) vs no Gain. *P*-value for each analysis was produced by log-rank test.

Supplementary Figure 7

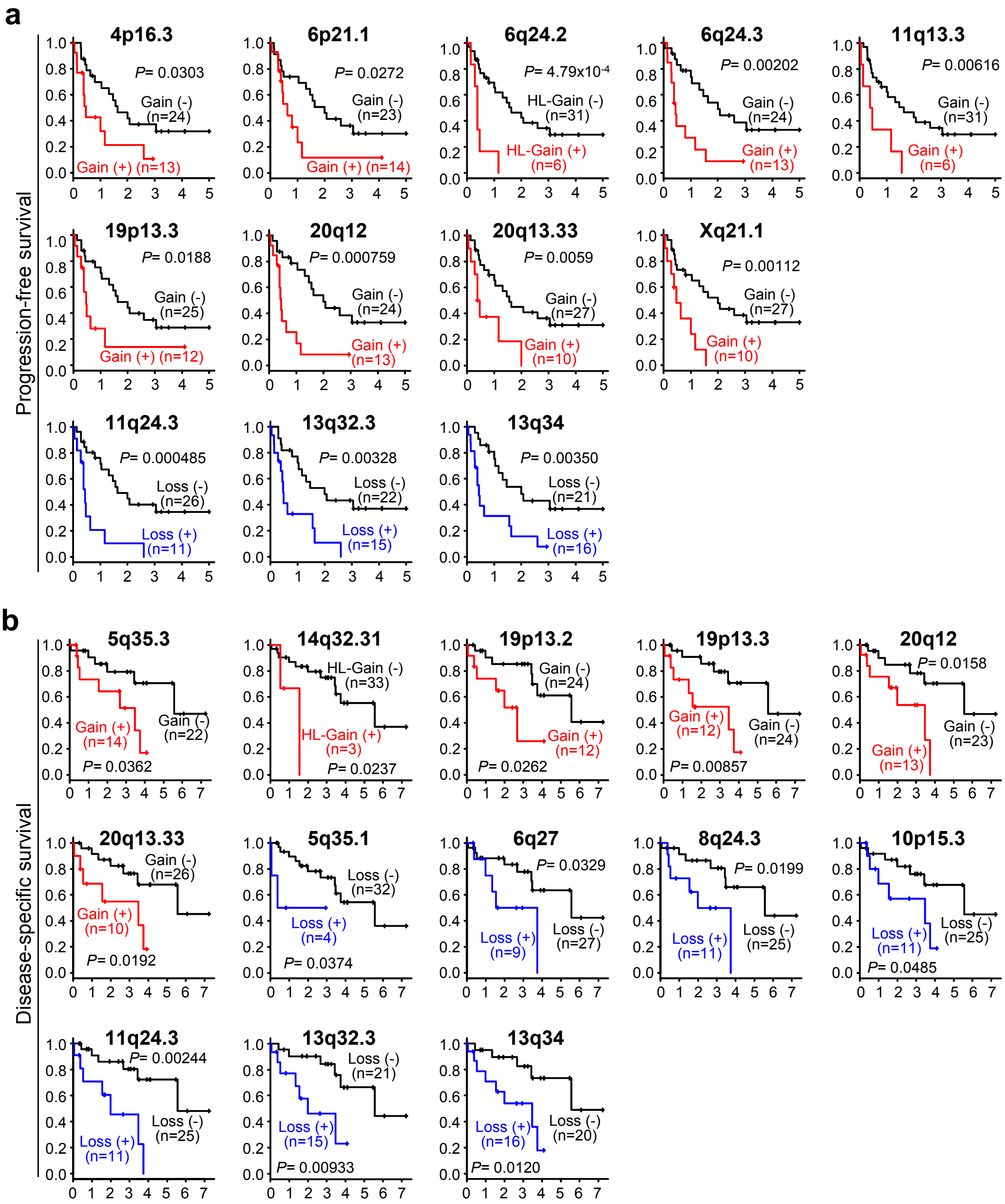

Supplementary Figure 7. Association of additional SCNA regions with progression-free (a) and disease-specific (b) survival for Cluster 1 DDLPS. Kaplan-Meier survival analysis was performed after stratifying the patients with Cluster 1 DDLPS by the status of recurrent SCNAs. Progression-free and disease-specific survival curves were analyzed by Kaplan-Meier methods. SCNAs with gain were stratified by two criteria: high-level gain (HL-Gain) vs no HL-Gain (low-level or no gain), and Gain (high- or low-level gain) vs no Gain. *P*-value for each analysis was produced by log-rank test.

Supplementary Figure 8

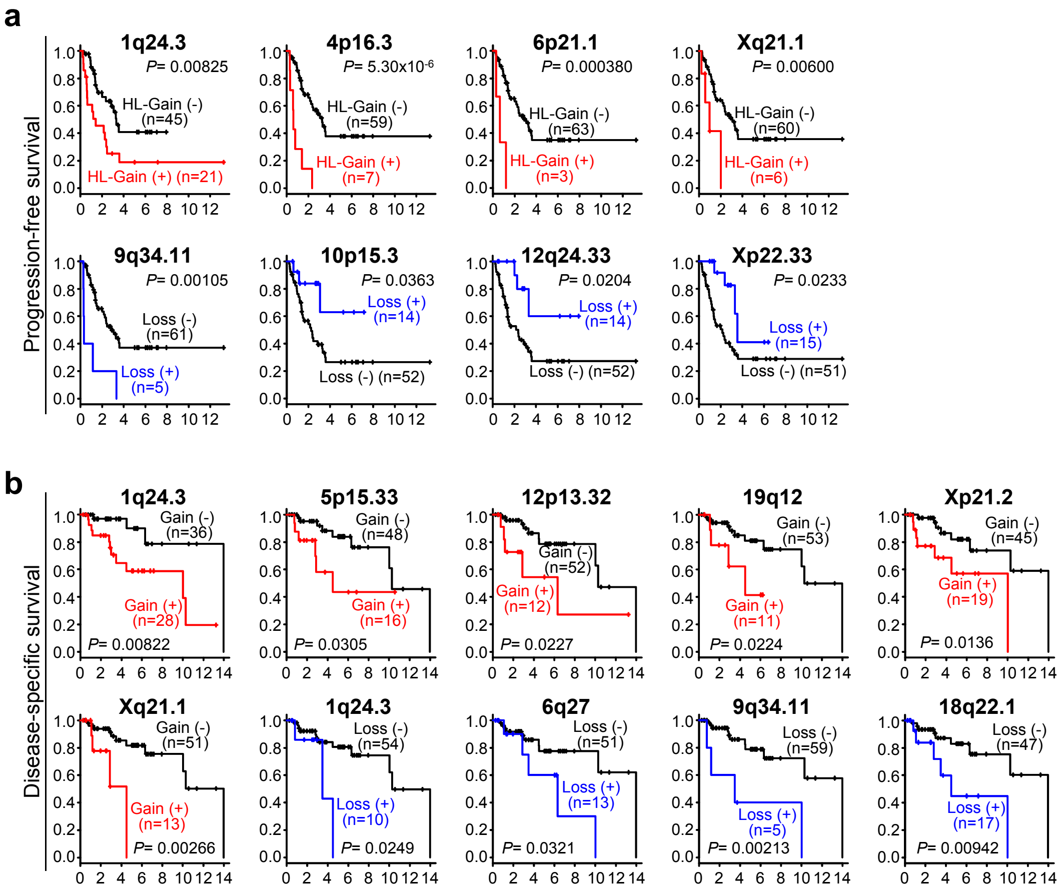

Supplementary Figure 8. Association of additional SCNA regions with progression-free (a) and disease-specific (b) survival for Cluster 2 DDLPS. Kaplan-Meier survival analysis was performed after stratifying the patients with Cluster 2 DDLPS by the status of recurrent SCNAs. Progression-free and disease-specific survival curves were analyzed by Kaplan-Meier methods. SCNAs with gain were stratified by two criteria: high-level gain (HL-Gain) vs no HL-Gain (low-level or no gain), and Gain (high- or low-level gain) vs no Gain. *P*-value for each analysis was produced by log-rank test.

Supplementary Figure 9

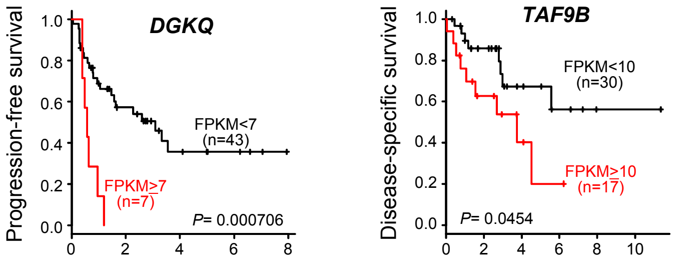

Supplementary Figure 9. Association of expression level of *DGKQ* and *TAF9B* with clinical prognosis in the TCGA cohort. Kaplan-Meier survival analysis was performed after stratifying the patients according to the expression of DGKQ and TAF9B, which were identified in **Supplementary Table 11**. Stratification criteria, determined by the frequency of copy-number gain of the gene, was described in each panel. *P*-value for each analysis was produced by log-rank test.

Supplementary Figure 10

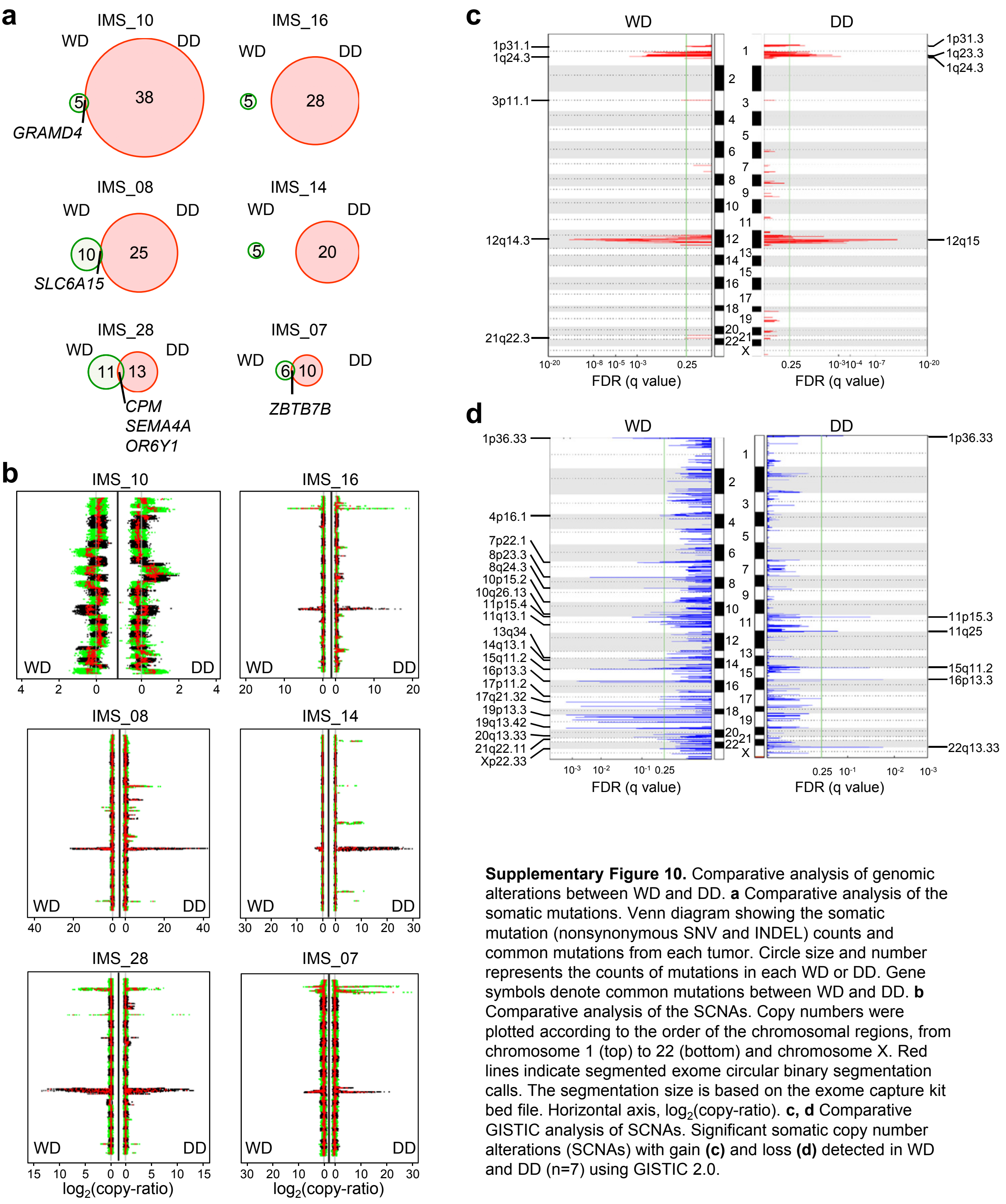

Supplementary Figure 10. Comparative analysis of genomic alterations between WD and DD. **a** Comparative analysis of the somatic mutations. Venn diagram showing the somatic mutation (nonsynonymous SNV and INDEL) counts and common mutations from each tumor. Circle size and number represents the counts of mutations in each WD or DD. Gene symbols denote common mutations between WD and DD. **b** Comparative analysis of the SCNAs. Copy numbers were plotted according to the order of the chromosomal regions, from chromosome 1 (top) to 22 (bottom) and chromosome X. Red lines indicate segmented exome circular binary segmentation calls. The segmentation size is based on the exome capture kit bed file. Horizontal axis, $\log_2$(copy-ratio). **c, d** Comparative GISTIC analysis of SCNAs. Significant somatic copy number alterations (SCNAs) with gain **(c)** and loss **(d)** detected in WD and DD (n=7) using GISTIC 2.0.

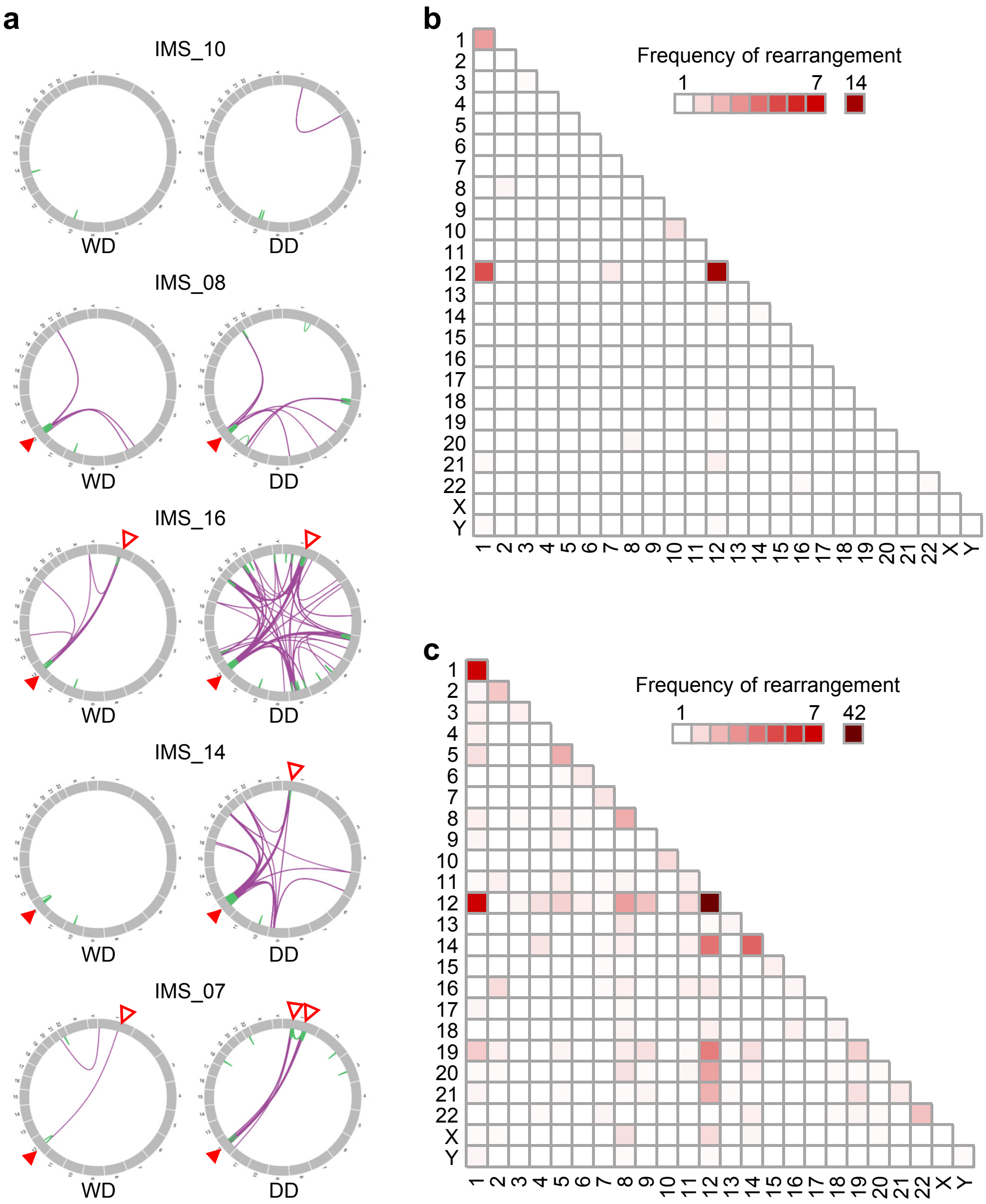

Supplementary Figure 11. Comparative analysis of the structural variants (SVs) between WD and DD. **a** SVs from WD and DD samples. Circos plots represent the inter- (purple) and intra-(green) chromosomal structural variants for WD (left) and DD (right). **b, c** Frequency of inter- and intra-chromosomal rearrangement in WD **(b)** and DD **(c).** Each box in the heatmap shows mean frequency of inter- or intra-chromosomal rearrangement. The frequency of chromosomal rearrangements in each sample was determined using Genomon Fusion to analysis RNA sequencing results from six matched pairs of WD and DD.

Supplementary Figure 12

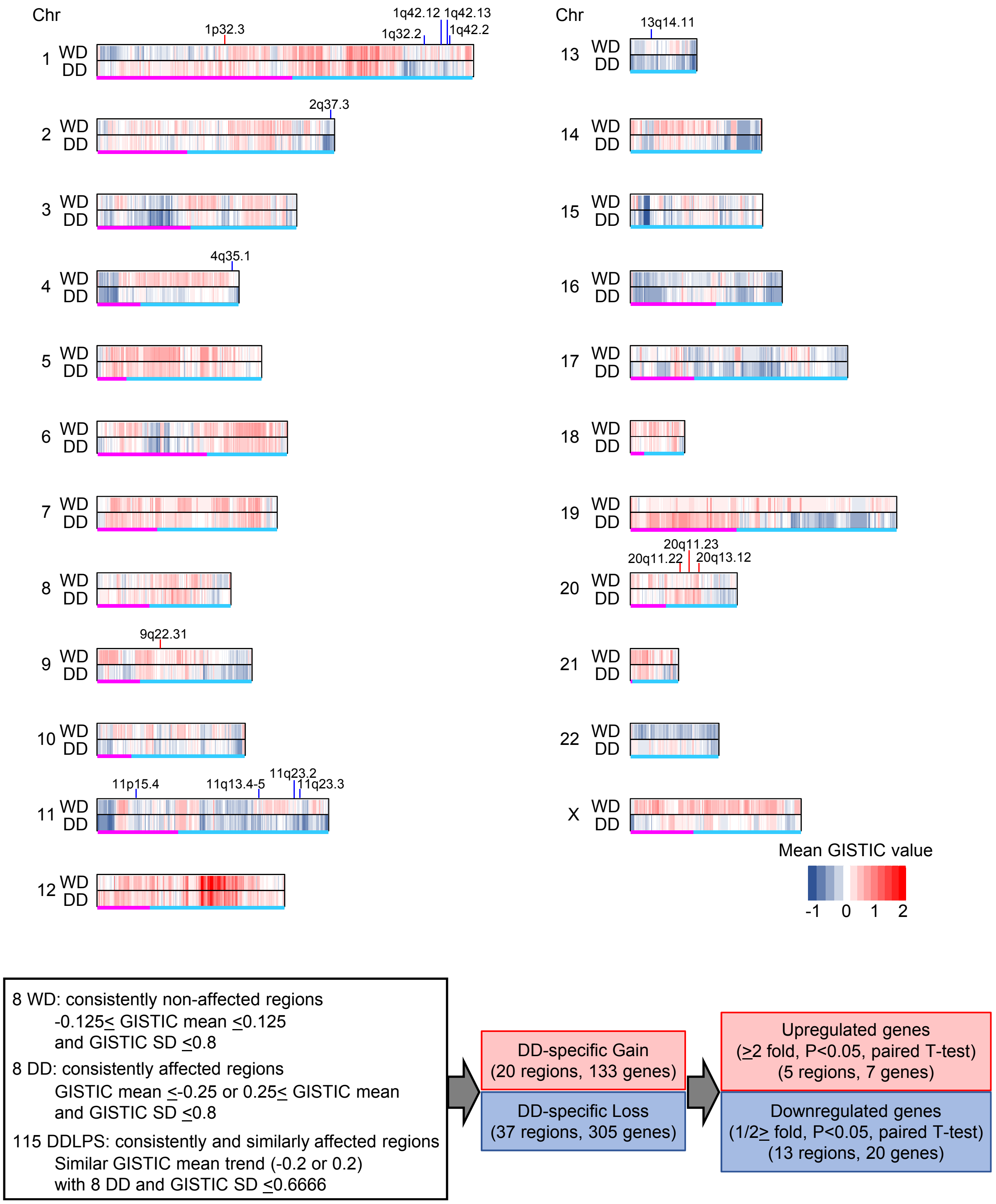

Supplementary Figure 12. Comparative genome-wide SCNA analysis between WD and DD. Vertical heat-colored lines represent mean GISTIC value of each gene from seven paired WD and DD. Regions with DD-specific SCNA were described with red (gain) and blue (loss) lines at each chromosome.

Supplementary Figure 13

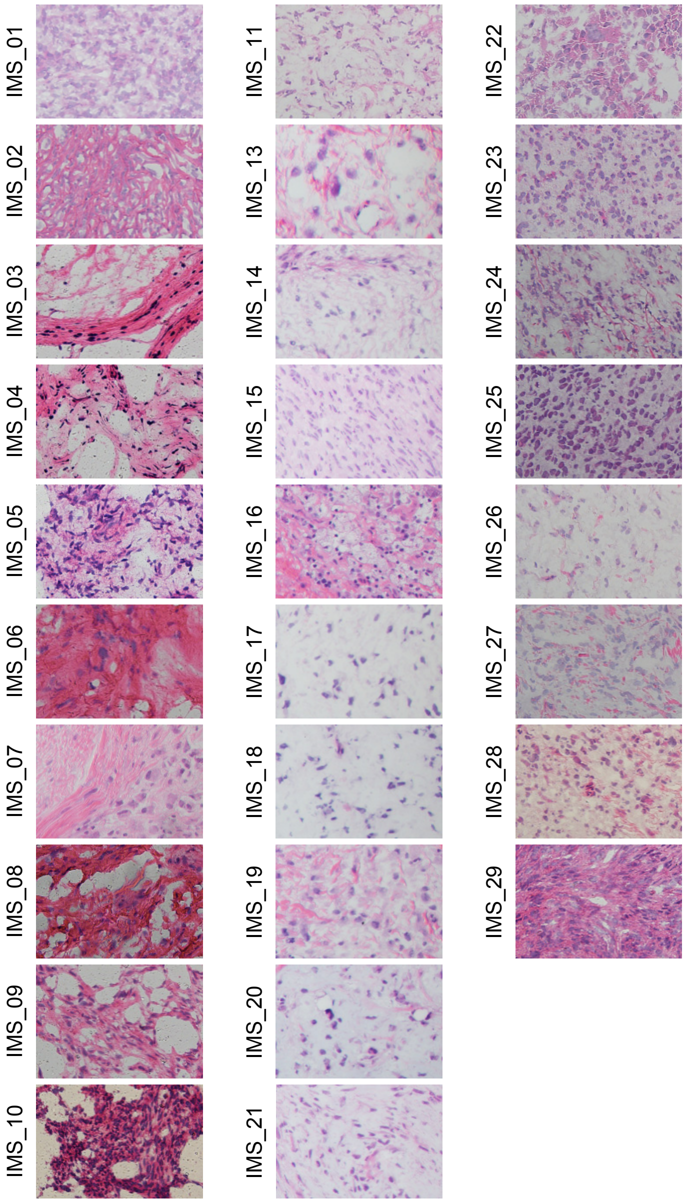

Supplementary Figure 13. HE staining of frozen DDLPS samples. 28 frozen DDLPS samples from JSGC-IMSUT were sectioned and stained with hematoxylin and eosin to validate pathological diagnosis and to examine tumor content.

Supplementary Table 1. Clinical characteristics of patients with DDLPS for each cohort

Features	JSGC (n=21)	NCC (n=37)	TCGA (n=50)
Male Sex, n (%)	13 (61.9)	29 (78.3)	33 (66.0)
Age at diagnosis (y)	66.2 ±14.0	60.2 ±11.3	63.0 ±12.8
Primary site, n (%)			
Retroperitoneum or abdomen	7 (33.3)	29 (78.4)	43 (86.0)
Extremity	14 (66.7)	6 (16.2)	5 (10.0)
Chest wall or back	0 (0.0)	2 (5.4)	2 (4.0)
Tumor size, n (%)			
10 cm ≥	5 (25.0)	11 (29.7)	9 (18.4)
10 cm <	15 (75.0)	26 (70.3)	40 (81.6)
Unknown	1 -	0 -	1 -
Local Treatment, n (%)			
Surgery-only	20 (95.2)	32 (86.5)	45 (90.0)
Surgery with adjuvant radiation	1 (4.8)	3 (8.1)	5 (10.0)
Radiation	0 (0.0)	1 (2.7)	0 (0.0)
Heavy ion	0 (0.0)	1 (2.7)	0 (0.0)
Surgical margin, n (%)			
R0	9 (42.9)	9 (24.3)	22 (44.0)
R1	12 (57.1)	22 (59.5)	24 (48.0)
R2	0 (0.0)	0 (0.0)	3 (6.0)
RX	0 (0.0)	4 (10.8)	1 (2.0)
Not applicable	0 -	2 -	0 -

Supplementary Table 2. Recurrent inter-chromosomal fusion genes

Chromosomes		Genes		Ratio (%)
15	7	*C15orf57*	*CBX3*	10/101 (9.9%)
1	12	*DNM3os*	*CTDSP2*	5/101 (5.0%)
1	2	*DNM3os*	*CTDSP1*	3/101 (3.0%)

Supplementary Table 3. Partner genes of *DNM3OS* fusions in DDLPS

ID	Gene 1	Gene 2	Breakpoint 1*	Breakpoint 2*	Supporting reads
NCC_05	*CTDSP2*	*DNM3OS*	Chr12:58220129	Chr1: 172113577	12
NCC_04	*CTDSP2*	*DNM3OS*	Chr12:58220129	Chr1: 172113577	8
IMS_16	*CTDSP2*	*DNM3OS*	Chr12:58220129	Chr1: 172113577	4
NCC_23	*CTDSP2*	*DNM3OS*	Chr12:58220129	Chr1: 172113577	4
NCC_18	*CTDSP2*	*DNM3OS*	Chr12:58220129	Chr1: 172113577	3
NCC_05	*CTDSP1*	*DNM3OS*	Chr2:219267128	Chr1: 172113577	6
NCC_20	*CTDSP1*	*DNM3OS*	Chr2:219267128	Chr1: 172113577	4
IMS_16	*CTDSP1*	*DNM3OS*	Chr2:219267128	Chr1: 172113577	3
NCC_24	*CPM*	*DNM3OS*	Chr12:69326458	Chr1: 172113577	5
IMS_17	*UTP20*	*DNM3OS*	Chr12:101761754	Chr1: 172113577	6
NCC_20	*ENST00000551075*	*DNM3OS*	Chr12:92299713	Chr1: 172113577	3

* Breakpoint positions were referred to hg19 sequence.

Supplementary Table 4. Cox regression analysis of progression-free (**a**) and disease-specific (**b**) survival with somatic copy number alterations

**a.** Progression-free surivival		**Univariate**			**Multivariate**		
Region	SCNA status	**HR**	**(95% CI)**	***P* value**	**HR**	**(95% CI)**	***P* value**
1p32.1	Gain (vs. no gain)	1.76	(1.06-2.94)	0.0301*	1.43	(0.77-2.66)	0.260
4p16.3	HL-gain (vs. LL- or no gain)	3.72	(1.86-7.45)	2.03×10^{-4}**	4.68	(2.03-10.76)	2.82×10^{-4}**
5p15.33	Gain (vs. no gain)	1.93	(1.15-3.23)	0.0122*	1.12	(0.60-2.09)	0.7170
6p21.1	HL-gain (vs. LL- or no gain)	3.09	(1.45-6.59)	3.54×10^{-3}**	2.57	(1.06-6.20)	0.0362*
20q12	Gain (vs. no gain)	1.84	(1.10-3.07)	0.0196*	0.95	(0.48-1.85)	0.870
Xp21.2	Gain (vs. no gain)	1.84	(1.10-3.08)	0.0213*	0.96	(0.49-1.91)	0.917
Xq21.1	Gain (vs. no gain)	2.71	(1.49-4.92)	1.07×10^{-3}**	2.87	(1.31-6.32)	8.65×10^{-3}**
2q37.3	Loss (vs. no loss)	1.74	(1.02-2.98)	0.0417*	0.89	(0.41-1.93)	0.7640
9p21.2	Loss (vs. no loss)	1.66	(1.00-2.76)	0.0517	1.15	(0.63-2.12)	0.6440
9q34.11	Loss (vs. no loss)	3.16	(1.59-6.27)	1.03×10^{-3}**	3.27	(1.28-8.35)	0.0135*
13q34	Loss (vs. no loss)	1.91	(1.15-3.20)	0.0131*	0.90	(0.36-2.22)	0.815
16q24.3	Loss (vs. no loss)	1.82	(1.00-3.31)	0.0494*	1.33	(0.56-3.20)	0.518

**b.** Disease-specific surivival		**Univariate**			**Multivariate**		
Region	SCNA status	**HR**	**(95% CI)**	***P* value**	**HR**	**(95% CI)**	***P* value**
1p32.1	Gain (vs. no gain)	2.95	(1.32-6.60)	0.00854**	2.66	(1.03-6.88)	0.0431*
5p15.33	Gain (vs. no gain)	2.18	(1.01-4.70)	0.0477*	0.91	(0.341-2.43)	0.852
5q35.3	Gain (vs. no gain)	2.74	(1.23-6.11)	0.0137*	1.14	(0.40-3.25)	0.807
19p13.3	Gain (vs. no gain)	2.59	(1.15-5.86)	0.0220*	1.53	(0.47-4.99)	0.480
19q12	Gain (vs. no gain)	2.94	(1.28-6.77)	0.0112*	1.66	(0.54-5.12)	0.379
Xq21.1	Gain (vs. no gain)	3.88	(1.59-9.44)	0.00284**	1.57	(0.45-5.51)	0.479
6q27	Loss (vs. no loss)	3.24	(1.47-7.17)	0.00371**	1.56	(0.48-5.05)	0.456
9p24.3	Loss (vs. no loss)	2.19	(1.02-4.67)	0.0436*	1.11	(0.24-5.06)	0.894
9p21.2	Loss (vs. no loss)	2.72	(1.26-5.87)	0.0110*	1.31	(0.26-6.63)	0.745
9q34.11	Loss (vs. no loss)	3.90	(1.63-9.38)	0.00233**	2.75	(0.62-12.25)	0.185
11p15.5	Loss (vs. no loss)	2.17	(1.01-4.67)	0.0479*	0.88	(0.24-3.20)	0.848
11q24.3	Loss (vs. no loss)	2.17	(1.01-4.68)	0.0486*	0.79	(0.23-2.67)	0.706
13q32.3	Loss (vs. no loss)	2.26	(1.04-4.89)	0.0390*	1.74	(0.43-7.14)	0.441
13q34	Loss (vs. no loss)	2.85	(1.31-6.19)	0.00819**	0.75	(0.13-4.30)	0.746
18q22.1	Loss (vs. no loss)	2.93	(1.34-6.42)	0.00720**	1.18	(0.39-3.58)	0.773

Results are presented for the univariate and multivariate Cox-regression analysis for progression-free and disease-specific survival. Kaplan-Meier survival curves according to the SCNA regions are shown in **Supplementary Fig. 6**. CI, confidence interval; HR, hazard ratio; * P<0.05, ** P<0.01

Supplementary Table 5. Cox regression analysis of progression-free survival with clinical variables and SCNA regions in DDLPS

Progression-free survival	Univariate			Multivariate		
	HR	(95% CI)	*P* value	HR	(95% CI)	*P* value
Primary tumor site						
Trunk (vs Extremity)†	5.22	(2.08-13.10)	4.29×10^{-4}**	4.19	(1.48-11.84)	6.99×10^{-3}**
Surgical margin						
(R2, R1, R0)	2.25	(1.33-3.81)	2.524×10^{-3}**	1.33	(0.711-2.50)	0.370
SCNA regions‡						
4p16.3 HL-gain (vs. LL- or no gain)	3.72	(1.86-7.45)	2.03×10^{-4}**	3.82	(1.60-9.11)	2.50×10^{-3}**
6p21.1 HL-gain (vs. LL- or no gain)	3.09	(1.45-6.59)	3.54×10^{-3}**	4.19	(1.74-10.08)	1.41×10^{-3}**
Xq21.1 Gain (vs. no gain)	2.71	(1.49-4.92)	1.07×10^{-3}**	3.05	(1.61-5.76)	6.20×10^{-4}**
9q34.11 Loss (vs. no loss)	3.16	(1.59-6.27)	1.03×10^{-3}**	3.37	(1.48-7.68)	3.83×10^{-3}**

CI, confidence interval; HR, hazard ratio; * P<0.05, ** P<0.01; † Trunk includes abdomen, retroperitoneum, chest wall and back, and Extremity includes extremity, shoulder, and girdle; ‡ SCNA regions were selected, based on the results shown in **Supplementary Table 4a**.

Supplementary Table 6. Cox regression analysis of progression-free (a) and disease-specific (b) survival with SCNA in Cluster 1 DDLPS

**a.** Progression-free surivival		Univariate			Multivariate		
Region	SCNA status	HR	(95% CI)	*P* value	HR	(95% CI)	*P* value
4p16.3	Gain (vs. no gain)	2.42	(1.06-5.53)	0.0359*	0.71	(0.20-2.50)	0.595
6p21.1	Gain (vs. no gain)	2.55	(1.08-5.98)	0.0321*	0.81	(0.18-3.55)	0.775
6q24.2	HL-gain (vs. LL- or no gain)	5.21	(1.86-14.58)	$1.66x10^{-3}$**	4.28	(0.30-60.76)	0.283
6q24.3	Gain (vs. no gain)	3.46	(1.50-7.97)	$3.50x10^{-3}$**	1.64	(0.31-8.59)	0.556
11q13.3	Gain (vs. no gain)	3.53	(1.35-9.24)	0.0104*	0.33	(0.05-2.31)	0.262
19p13.3	Gain (vs. no gain)	2.69	(1.14-6.31)	0.0234*	0.59	(0.11-3.16)	0.540
20q12	Gain (vs. no gain)	3.95	(1.69-9.25)	$1.57x10^{-3}$**	4.48	(0.63-31.74)	0.134
20q13.33	Gain (vs. no gain)	3.30	(1.35-8.11)	$9.14x10^{-3}$**	1.64	(0.342-7.86)	0.537
Xq21.1	Gain (vs. no gain)	4.04	(1.63-10.01)	$2.52x10^{-3}$**	1.93	(0.50-7.48)	0.342
11q24.3	Loss (vs. no loss)	4.07	(1.75-9.49)	$1.15x10^{-3}$**	0.76	(0.15-3.76)	0.736
13q32.3	Loss (vs. no loss)	3.27	(1.43-7.51)	$5.19x10^{-3}$**	9.23	(1.06-80.50)	0.0444*
13q34	Loss (vs. no loss)	3.18	(1.40-7.20)	$5.54x10^{-3}$**	0.39	(0.04-3.59)	0.409

**b.** Disease-sepcific surivival		Univariate			Multivariate		
Region	SCNA status	HR	(95% CI)	*P* value	HR	(95% CI)	*P* value
5q35.3	Gain (vs. no gain)	3.21	(1.01-10.19)	0.0473*	1.84	(0.21-16.02)	0.579
14q32.31	Gain (vs. no gain)	4.28	(1.02-17.97)	0.0469*	0.66	(0.05-9.06)	0.756
19p13.2	Gain (vs. no gain)	3.59	(1.09-11.79)	0.0355*	3.61	(0.73-17.99)	0.117
19p13.3	Gain (vs. no gain)	4.20	(1.32-13.42)	0.0153*	1.87	(0.06-55.37)	0.717
20q12	Gain (vs. no gain)	3.89	(1.20-12.66)	0.0240*	0.46	(0.01-15.22)	0.667
20q13.33	Gain (vs. no gain)	3.58	(1.15-11.21)	0.0282*	2.36	(0.24-23.52)	0.466
5q35.1	Loss (vs. no loss)	4.47	(0.91-21.98)	0.0657	7.71	(0.50-119.4)	0.144
6q27	Loss (vs. no loss)	3.37	(1.04-10.94)	0.0436*	2.71	(0.23-32.23)	0.429
8q24.3	Loss (vs. no loss)	3.76	(1.15-12.30)	0.0285*	0.90	(0.03-24.80)	0.948
10p15.3	Loss (vs. no loss)	2.98	(0.96-9.32)	0.0600	0.24	(0.01-4.38)	0.335
11q24.3	Loss (vs. no loss)	5.15	(1.60-16.63)	$6.11x10^{-3}$**	2.98	(0.04-199.4)	0.611
13q32.3	Loss (vs. no loss)	4.33	(1.32-14.20)	0.0158*	3.61	(0.26-49.38)	0.336
13q34	Loss (vs. no loss)	4.21	(1.25-14.16)	0.0203*	0.82	(0.02-31.92)	0.916

Results are presented for the univariate and multivariate Cox-regression analysis for progression-free and disease-specific survival. Kaplan-Meier survival curves according to the SCNA regions are shown in **Supplementary Fig. 7**. CI, confidence interval; HR, hazard ratio; * P<0.05, ** P<0.01

Supplementary Table 7. Cox regression analysis of progression-free (a) and disease-specific (b) survival with SCNA in Cluster 2 DDLPS

**a.** Progression-free surivival		Univariate			Multivariate		
Region	SCNA status	HR	(95% CI)	*P* value	HR	(95% CI)	*P* value
1q24.3	HL-gain (vs. LL- or no gain)	2.39	(1.23-4.66)	0.0104*	2.49	(1.19-5.21)	0.0152*
4p16.3	HL-gain (vs. LL- or no gain)	6.04	(2.51-14.53)	$2.45x10^{-3}$**	5.13	(1.80-14.64)	$2.27x10^{-3}$**
6p21.1	HL-gain (vs. LL- or no gain)	7.07	(2.01-24.86)	$5.99x10^{-5}$**	3.08	(0.55-17.42)	0.202
Xq21.1	HL-gain (vs. LL- or no gain)	4.11	(1.38-12.27)	0.0112*	18.25	(4.81-69.26)	$1.97x10^{-5}$**
9q34.11	Loss (vs. no loss)	4.35	(1.67-11.35)	$2.68x10^{-3}$**	20.32	(4.55-90.76)	$8.04x10^{-5}$**
10p15.3	Loss (vs. no loss)	0.30	(0.09-0.99)	0.0482*	0.47	(0.13-1.74)	0.257
12q24.33	Loss (vs. no loss)	0.27	(0.08-0.88)	0.0305*	0.18	(0.05-0.74)	0.0167*
Xp22.33	Loss (vs. no loss)	0.32	(0.11-0.90)	0.0316*	0.32	(0.10-0.96)	0.0422*

**b.** Disease-sepcific surivival		Univariate			Multivariate		
Region	SCNA status	HR	(95% CI)	*P* value	HR	(95% CI)	*P* value
1q24.3	Gain (vs. no gain)	4.77	(1.33-17.14)	0.0166*	4.89	(0.91-26.18)	0.0640
5p15.33	Gain (vs. no gain)	3.07	(1.06-8.93)	0.0396*	3.71	(0.74-18.68)	0.112
12p13.32	Gain (vs. no gain)	3.35	(1.11-10.11)	0.0316*	3.96	(0.80-19.62)	0.0915
19q12	Gain (vs. no gain)	3.81	(1.11-13.07)	0.0337*	0.48	(0.06-4.01)	0.501
Xp21.2	Gain (vs. no gain)	3.63	(1.21-10.84)	0.0211*	0.78	(0.18-3.29)	0.733
Xq21.1	Gain (vs. no gain)	5.90	(1.61-21.56)	$7.33x10^{-3}$**	3.57	(0.35-36.63)	0.284
1q24.3	Loss (vs. no loss)	4.14	(1.08-15.93)	0.0386*	2.62	(0.45-15.40)	0.286
6q27	Loss (vs. no loss)	3.18	(1.04-9.72)	0.0425*	0.94	(0.21-4.18)	0.934
9q34.11	Loss (vs. no loss)	5.36	(1.62-17.74)	$5.94x10^{-4}$**	2.67	(0.58-12.18)	0.206
18q22.1	Loss (vs. no loss)	3.84	(1.29-11.45)	0.0159*	2.36	(0.40-13.98)	0.345

Results are presented for the univariate and multivariate Cox-regression analysis for progression-free and disease-specific survival. Kaplan-Meier survival curves according to the SCNA regions are shown in **Supplementary Fig. 8**. CI, confidence interval; HR, hazard ratio; * P<0.05, ** P<0.01

Supplementary Table 8. Genes with varied expression dependently modulated by SCNAs*

Gene	Cytogenetic band	Jonckheere-Terpstra test		
		TCGA	IMS	NCC
JUN	1p32.1	9.10×10^{-4}	0.0438	3.25×10^{-3}
DNM3	1q24.3	1.66×10^{-6}	0.0198	1.68×10^{-6}
DNM3OS	1q24.3	1.05×10^{-10}	0.0281	2.13×10^{-4}
TAF9B	Xq21.1	1.54×10^{-4}	0.0484	5.54×10^{-3}
DGKQ	4p16.3	3.16×10^{-4}	0.0440	7.75×10^{-4}
STX18	4p16.3	1.62×10^{-6}	0.0141	6.26×10^{-5}

Jonckheere-Terpstra test was performed between the samples with any gain vs no gain. Genes with less than 0.05 of p-value in all three cohorts were listed.

* SCNAs, significantly associated with clinical outcomes, were selected.

Supplementary Table 9. Gene sets*, significantly enriched in DD (a) and WD (b)

**a.** Gene sets enriched in DD	ES	NES	NOM p-val	FDR q-val
G2M_CHECKPOINT	0.73	1.90	0.000	0.001
E2F_TARGETS	0.76	1.81	0.000	0.007
SPERMATOGENESIS	0.42	1.48	0.032	0.217

**b.** Gene sets enriched in WD	ES	NES	NOM p-val	FDR q-val
ADIPOGENESIS	-0.71	-1.91	0.000	0.025
MYOGENESIS	-0.55	-1.83	0.003	0.046
BILE_ACID_METABOLISM	-0.50	-1.82	0.000	0.031
XENOBIOTIC_METABOLISM	-0.51	-1.80	0.000	0.034
ESTROGEN_RESPONSE_EARLY	-0.45	-1.79	0.000	0.032
FATTY_ACID_METABOLISM	-0.54	-1.69	0.028	0.071
UV_RESPONSE_DN	-0.43	-1.62	0.009	0.106
ANGIOGENESIS	-0.49	-1.59	0.017	0.117
HEME_METABOLISM	-0.36	-1.53	0.010	0.151
ESTROGEN_RESPONSE_LATE	-0.36	-1.52	0.006	0.147
NOTCH_SIGNALING	-0.42	-1.47	0.010	0.186
APICAL_SURFACE	-0.39	-1.43	0.034	0.212

* Hallmark gene sets were applied for the analysis

Supplementary Table 10. Up- and down-regulated genes at the DD-specific copy-number gain (**a**) and loss (**b**) regions*

**a.** Upregulated genes at gain regions	Cytoband	WD components Mean FPKM	WD components S.D.	DD components Mean FPKM	DD components S.D.	P-value (-log10)	Ratio (log2)
ORC1	1p32.3	0.26	0.14	1.66	1.15	1.92	2.68
WFDC3	20q13.12	1.46	1.00	5.22	4.03	1.63	1.83
LRRC42	1p32.3	8.36	1.13	20.82	12.52	1.55	1.32
UBE2C	20q13.12	4.33	3.17	56.73	55.91	1.49	3.71
SPAG4	20q11.22	1.42	0.65	8.01	7.09	1.45	2.49
ROR2	9q22.31	2.07	2.19	6.36	3.98	1.39	1.62
NKAIN1	1p35.2	0.63	0.47	1.93	1.50	1.37	1.61

**b.** Downregulated genes at loss regions	Cytoband	WD components Mean FPKM	WD components S.D.	DD components Mean FPKM	DD components S.D.	P-value (-log10)	Ratio (log2)
SIDT2	11q23.3	27.10	7.65	10.77	5.54	4.51	-1.33
UVRAG	11q13.5	12.63	3.40	5.00	2.28	4.16	-1.34
MGLL	3q21.3	27.89	8.47	12.26	5.14	4.05	-2.24
FOXO1	13q14.11	15.91	3.39	4.80	2.43	3.84	-1.73
EPHX1	1q42.12	169.30	53.82	41.06	24.38	2.88	-2.04
G0S2	1q32.2	1201.34	579.28	45.37	35.38	2.61	-4.73
SCUBE2	11p15.4	8.66	3.76	2.76	1.29	2.59	-1.65
ABTB1	3q21.3	85.80	19.62	18.19	7.82	2.45	-1.19
DGAT2	11q13.5	70.83	40.01	1.72	1.81	2.40	-5.36
C1orf198	1q42.2	37.96	15.26	14.57	2.54	2.39	-1.38
GDPD5	11q13.4	19.40	10.05	3.12	1.29	2.35	-2.64
ACSL1	4q35.1	212.11	101.31	25.13	12.79	2.35	-3.08
GJC2	1q42.13	1.88	0.86	0.93	0.61	2.17	-1.02
TMPRSS5	11q23.2	1.77	1.33	0.37	0.54	2.17	-2.25
WNT11	11q13.5	38.35	13.61	11.83	8.72	2.16	-1.70
SCN4B	11q23.3	13.59	7.37	3.20	1.87	1.93	-2.09
MAP6	11q13.5	0.69	0.41	0.14	0.14	1.76	-2.25
PLXNA2	1q32.2	5.84	3.16	2.18	1.25	1.65	-1.42
HSD11B1	1q32.2	12.78	7.57	5.65	5.26	1.46	-1.18
AGXT	2q37.3	0.54	0.56	0.01	0.02	1.43	-5.54

DD-specific copy-number-gain and -loss regions were identified by volcano plots (**Fig. 4 g, h**)

Supplementary Table 11. Cox regression analysis of progression-free (**a**) and disease-specific (**b**) survival with clinical variables and genomic clustering

a. Progression-free survival		Univariate			Multivariate	
	HR	(95% CI)	*P* value	HR	(95% CI)	*P* value
Primary tumor site						
Trunk (vs Extremity)†	5.22	(2.08-13.10)	$4.29x10^{-4}$**	4.19	(1.55-11.34)	$4.81x10^{-3}$**
Surgical margin						
(R2, R1, R0)	2.25	(1.33-3.81)	$2.52x10^{-3}$**	1.56	(0.82-2.97)	0.175
Age at diagnosis	0.99	(0.98-1.02)	0.620	0.99	(0.97-1.01)	0.264
Gender						
Female (vs Male)	0.80	(0.44-1.43)	0.450	0.91	(0.49-1.68)	0.757
Size						
10 cm < (vs 10 cm ≥)	0.62	(0.35-1.11)	0.106	0.62	(0.33-1.18)	0.147
Genomic cluster						
Cluster1 (vs 2)	1.82	(1.08-3.04)	0.0234*	2.17	(1.19-3.96)	$1.17x10^{-2}$*
Cluster3 (vs 2)	0.399	(0.55-2.92)	0.365	0.99	(0.13-7.83)	0.996

b. Disease-specific survival		Univariate			Multivariate	
	HR	(95% CI)	*P* value	HR	(95% CI)	*P* value
Primary tumor site						
Trunk (vs Extremity)†	8.14	(1.10-60.06)	0.0397*	9.51	(1.21-74.81)	$3.24x10^{-2}$*
Surgical margin						
(R2, R1, R0)	2.70	(1.16-6.26)	0.0207*	1.68	(0.68-4.15)	0.264
Age at diagnosis	1.03	(1.00-1.07)	0.0816	1.05	(1.01-1.09)	$1.81x10^{-2}$*
Gender						
Female (vs Male)	1.32	(0.57-3.06)	0.523	1.43	(0.58-3.51)	0.432
Size						
10 cm < (vs 10 cm ≥)	0.54	(0.24-1.22)	0.139	0.49	(0.19-1.22)	0.124
Genomic cluster						
Cluster1 (vs 2)	2.86	(1.28-6.39)	0.0104*	2.97	(1.23-7.19)	$1.57x10^{-2}$*
Cluster3 (vs 2)	$1.38x10^{-8}$	(0-Inf)	0.999	$1.89x10^{-7}$	(0-Inf)	0.998

Results are presented for the univariate and multivariate Cox-regression analysis for progression-free and disease-specific survival, using clinical measures and genomic cluster. Kaplan-Meier survival curves according to the SCNA regions are shown in Fig. 3. CI, confidence interval; HR, hazard ratio; * P<0.05, ** P<0.01; †Trunk includes abdomen, retroperitoneum, chest wall and back, and Extremity includes extremity, shoulder, and girdle.
