# Supplementary material for: Integrated exome and RNA sequencing of dedifferentiated liposarcoma
Source: Nat Commun. 2019 Dec 12;10:5683. doi: 10.1038/s41467-019-13286-z (PMC6908635; doi:10.1038/s41467-019-13286-z)
Supplement: Supplementary file 3 — Description of Additional Supplementary Files [file 41467_2019_13286_MOESM3_ESM.pdf]

Description of Additional Supplementary Files

File Name: Supplementary Data 1
Description: Genes and chromosomal regions with recurrent gain of copy number identified by GISTIC

File Name: Supplementary Data 2
Description: Genes and chromosomal regions with recurrent loss of copy number identified by GISTIC

File Name: Supplementary Data 3
Description: Genes with consistent copy-number gain or loss in Cluster 3
