# Supplementary material for: Integrated exome and RNA sequencing of dedifferentiated liposarcoma
Source: Nat Commun. 2019 Dec 12;10:5683. doi: 10.1038/s41467-019-13286-z (PMC6908635; doi:10.1038/s41467-019-13286-z)
Supplement: Supplementary file 7 — Reporting Summary [file 41467_2019_13286_MOESM7_ESM.pdf]

natureresearch

Corresponding author(s): Rui Yamaguchi, Hitoshi Ichikawa, Koichi Matsuda

Reporting Summary

Nature Research wishes to improve the reproducibility of the work that we publish. This form provides structure for consistency and transparency in reporting. For further information on Nature Research policies, see Authors & Referees and the Editorial Policy Checklist.

Statistical parameters

When statistical analyses are reported, confirm that the following items are present in the relevant location (e.g. figure legend, table legend, main text, or Methods section).

n/a	Confirmed	
☐	☒	The exact sample size (n) for each experimental group/condition, given as a discrete number and unit of measurement
☐	☒	An indication of whether measurements were taken from distinct samples or whether the same sample was measured repeatedly
☐	☒	The statistical test(s) used AND whether they are one- or two-sided<br>*Only common tests should be described solely by name; describe more complex techniques in the Methods section.*
☐	☒	A description of all covariates tested
☐	☒	A description of any assumptions or corrections, such as tests of normality and adjustment for multiple comparisons
☐	☒	A full description of the statistics including central tendency (e.g. means) or other basic estimates (e.g. regression coefficient) AND variation (e.g. standard deviation) or associated estimates of uncertainty (e.g. confidence intervals)
☐	☒	For null hypothesis testing, the test statistic (e.g. F, t, r) with confidence intervals, effect sizes, degrees of freedom and P value noted<br>*Give P values as exact values whenever suitable.*
☒	☐	For Bayesian analysis, information on the choice of priors and Markov chain Monte Carlo settings
☒	☐	For hierarchical and complex designs, identification of the appropriate level for tests and full reporting of outcomes
☐	☒	Estimates of effect sizes (e.g. Cohen's d, Pearson's r), indicating how they were calculated
☐	☒	Clearly defined error bars<br>*State explicitly what error bars represent (e.g. SD, SE, CI)*

Our web collection on statistics for biologists may be useful.

Software and code

Policy information about availability of computer code

Data collection	FASTQ data of exome and RNA sequencing was downloaded from [https://portal.gdc.cancer.gov/].<br>The Patient IDs in the manuscript were same as those in the published database.
Data analysis	Genomon (ver.2.0 and 2.2) for mutation call and fusion detection. GISTIC (ver.2.0.22) for copy number analysis. Tophat 2 (ver.2.1.0) and Cufflinks (ver. 2.2.1) for rna expression analysis.<br>R commander version 2.1-2 software were used for the survival analysis. A p-value of < 0.05 was considered statistically significant.

For manuscripts utilizing custom algorithms or software that are central to the research but not yet described in published literature, software must be made available to editors/reviewers upon request. We strongly encourage code deposition in a community repository (e.g. GitHub). See the Nature Research guidelines for submitting code & software for further information.

Data

Policy information about availability of data

All manuscripts must include a data availability statement. This statement should provide the following information, where applicable:

- Accession codes, unique identifiers, or web links for publicly available datasets
- A list of figures that have associated raw data
- A description of any restrictions on data availability

Sequencing FASTQ data files have been deposited at the Japanese Genotype-phenotype Archive (JGA, http://trace.ddbj.nig.ac.jp/jga), which is hosted by the DDBJ, under accession number JGAS00000000177.

Field-specific reporting

Please select the best fit for your research. If you are not sure, read the appropriate sections before making your selection.

- [x] Life sciences
- [ ] Behavioural & social sciences
- [ ] Ecological, evolutionary & environmental sciences

For a reference copy of the document with all sections, see nature.com/authors/policies/ReportingSummary-flat.pdf

Life sciences study design

All studies must disclose on these points even when the disclosure is negative.

Sample size	No explicit calculations were performed to determine sample size. Rather, we aimed to analyze all possible dedifferentiated liposarcoma samples.
Data exclusions	Samples with low quality or low quantity of DNA and RNA were excluded before the subsequent next-generation sequence. Patients with indefinite pathological diagnosis were also excluded from the study.<br>Somatic mutations, which didn't satisfy the following criteria, were excluded; more than 0.05 of mutation allele frequency in tumor tissues, less than 0.025 of mutation allele frequency in normal tissues, less than 0.001 of p-values by Fisher's exact test, more than 0.1 and less than 0.9 of strand ratio in tumor tissues.
Replication	All qPCR experiments were performed at least two times to assess reproducibility, which is described in Materials and Methods.
Randomization	The patients with DDLPS were categorized into three groups according to the status of copy-number alterations at 1q32.1 and 12q15, as shown in Figure 3.
Blinding	Genomic clustering of DDLPS patients was performed without knowledge of known prognostic status, such as age, size and primary site, which were only assessed after.

Reporting for specific materials, systems and methods

Materials & experimental systems

n/a	Involved in the study	
[x]	[ ]	Unique biological materials
[x]	[ ]	Antibodies
[x]	[ ]	Eukaryotic cell lines
[x]	[ ]	Palaeontology
[x]	[ ]	Animals and other organisms
[ ]	[x]	Human research participants

Methods

n/a	Involved in the study	
[x]	[ ]	ChIP-seq
[x]	[ ]	Flow cytometry
[x]	[ ]	MRI-based neuroimaging

Human research participants

Policy information about studies involving human research participants

Population characteristics	The characteristics of the research participants were described in Table 1.
Recruitment	Patients, diagnosed with DDPLS in collaborating hospitals, were recruited with the current study. The present protocols were reviewed and approved by the Ethics Committees of all participating institutions, including the Institute of Medical Science, the

University of Tokyo, the National Cancer Center, Japan, Tokyo Metropolitan Cancer and Infectious Diseases Center Komagome Hospital, Kyushu University, Osaka International Cancer Institute, Chiba Cancer Center, Nagoya University Graduate School of Medicine, Kanagawa Cancer Center, National Hospital Organization Hokkaido Cancer Center, and RIKEN Center for Integrative Medical Sciences. All of the participants were enrolled and anonymised after obtaining written informed consent.
